# HTLV-1 Is Also a Sexually Transmitted Infection

**DOI:** 10.3389/fpubh.2022.840295

**Published:** 2022-03-31

**Authors:** Mariana Martel, Eduardo Gotuzzo

**Affiliations:** ^1^Faculty of Medicine, Universidad Peruana Cayetano Heredia, Lima, Peru; ^2^Alexander von Humboldt Institute of Tropical Medicine, Cayetano Heredia National Hospital, Lima, Peru

**Keywords:** human *T*-lymphotropic virus 1, sexually transmitted diseases, risk factors, sex work, organization, world health

## Abstract

HTLV-1 is a retrovirus which causes diverse diseases in 10% of its infected population, significantly worsening their quality of life and mortality rate. Even though it is globally distributed and is endemic in many countries (including Peru), it is still highly neglected. It spreads through vertical, sexual and parenteral transmission. As no effective treatment against this virus exist, prevention is required to contain it. The World Health Organization published a technical report on the matter in 2021, with the collaboration of international HTLV-1 experts. However, neither the impact of sexual transmission (cause of the majority of adult cases and infection in non-endemic areas) nor its prevention were considered. Evidence is presented, which shows the magnitude of sexual transmission, its risk factors and preventive measures; hoping it will encourage health workers to help eradicate this infection.

## Introduction

Human *T*-cell lymphotropic virus 1 (HTLV-1) was the first human oncogenic retrovirus to be discovered 40 years ago (1). With clinical presentations, ranging from immunosuppression to excessive inflammation and the appearance of opportunistic infections, it was determined as the main cause of adult *T*-cell leukemia/lymphoma (ATL) and tropical spastic paraparesis or HTLV-1 associated myelopathy (TSP/HAM) (1). Of those infected, only 10% develop disease but have a high risk of complications and mortality (2).

According to outdated 9-yeard-old information, it affects more than 10 million people (3). But this has not been updated due to the lack of interest in HTVL-1. By being transmitted vertically (from mother to child through breastfeeding), sexually and parenterally (blood transfusion and intravenous drug users) (3), it is possible to reduce its incidence through prevention. This is of vital importance because there is no efficient treatment for its multiple clinical manifestations (1) and it is totally neglected wordlwide.

HTLV-1 has a global distribution: spanning Japan, the Caribbean Islands, South America, West and Central Africa, Romania, parts of the Middle East (Iran), and Central Australia (4). In Peru, it is estimated that this virus affects 1–2% of the population (5), with a recent systematic review and meta-analysis finding a pooled proportion of HTLV-1 of 2.34% in the general population of the country (95% CI; 1.96–2.75%; I^2^ = 95.51%) (6). A study carried out in the Quechua population from the provinces of Cangallo, Vilcashuaman, and Parinacochas found HTLV-1 in two of the three included villages (5). Additionally, the authors refer to a previous cohort of infected patients in Lima, of whom more than 50% where from this population (5). This suggests a high frequency of infection in the Andean region of Peru (5).

The prevalence of HTLV-1 is higher in women, who are more susceptible to TSP/HAM (1); which is associated with sexual transmission because it occurs mainly in people infected during adulthood (4). In contrast, men are more likely to suffer from ATL (1), of which the majority of cases are attributed to vertical (perinatal) infection (4). In order to prevent new HTLV-1 infections that could later end up in ATL, HTLV-1 screening of pregnant women and avoidance of lactation by carrier mothers was carried out in Japan. These measures managed to reduce the prevalence of this virus in the prefecture of Nagasaki from 7.2 to 1.4% in 30 years (4). However, the prevention of sexual transmission is not promoted internationally.

## Sexual Transmission of HTLV-1

Sexual transmission of HTLV-1 has been observed to be more efficient from male to female (1), presenting in Japan a transmission rate of 60.8% if the carrier is male (in stable partners for over 10 years); and one of 0.4% if the carrier is female (7). Similarly, a cohort carried out in Miyazaki found a rate of transmission 3.9 times higher in serodiscordant couples if the male was seropositive (8).

Cross-sectional data from the Miyazaki population strongly reinforce the likelihood of heterosexual transmission of HTLV-1, as men were more likely to be seropositive if their wives were positive and vice-versa (*p* < 0.001) (8), meaning it is highly possible that the transmission rate from women to men in HTLV-1 discordant couples has increased over the years.

This infection mechanism could be the most important within epidemiologically closed communities, such as the indigenous populations of Brazil; where there is an increase in the prevalence of HTLV-1 with age and a similar transmission from male to female and female to male (9).

In Salvador, epicenter of HTLV-1 in Brazil, a higher prevalence of the virus was seen in patients with a history of sexually transmitted infections (STIs) and older age, predominantly women, and there was an absence of infection in children under 13 years of age (10); all these being indicative of sexual transmission. A study in this area found that people with syphilis were 40 times more likely to be seropositive for HTLV-1. The risk factors associated with the transmission of the virus in this population were unprotected sex, multiple sexual partners, history of STIs, low level of education and low socioeconomic status (10). Risky sexual behaviors were hypothesized to stem from the cultural differences between developed and developing countries. For example, widespread use of condom in Japan could explain the predominance of vertical transmission in this region, compared to Brazil, where the sexual transmission predominates (10).

Extensive evidence suggests the importance of sexual transmission of HTLV-1. A cohort study of Japanese blood donors aged 16–69 years found a higher incidence in women (50–59 years) than in men (60–69 years) in endemic and non-endemic areas (11). However, men aged 20–29 years from metropolitan areas had a higher seroconversion rate than those from endemic areas. Thus, highlighting the probability of horizontal transmission between heterosexual couples, as opposed to vertical, which is believed to be the most common route of transmission in endemic areas (11). In Australia, it was thought that mother-to-child transmission could explain the number of cases, but a 2000–2013 cohort of indigenous children and adults found associations of HTLV-1 infection with increasing age, male sex, and previous STIs (12). There is even documentation of TSP/HAM developed by a female patient who was infected with HTLV-1 by her seropositive partner through sexual transmission, as other routes were dismissed during diagnosis (13).

Due to the similarity between the human immunodeficiency virus (HIV) and HTLV-1 transmission, multiple studies were done on HIV risk groups to define the groups susceptible to HTVL-1: female sex workers (FSW), men who have sex with men (MSM), STI patients and injecting drug users (14). HTLV-1 has been associated with coinfection with hepatitis B virus (HBV) and, to a lesser extent, HIV and syphilis (14). Patients infected with these STIs were considered responsible for HTLV-1 expansion into non-endemic areas. In Pará, Brazil, a study of 339 FSW determined the practice of unprotected sexual relations as a significant risk factor for HTLV-1 infection (adjusted OR/AOR 9.3; 95% CI; 4.9–14.2; *p* = 0.01) (15).

Before the appearance of current antiretroviral therapy (ART) against HIV, HTLV-1 was an influencing factor in the outcome of infected patients. A publication from the pre-ART era, described a not significant higher mortality and a shorter survival time in those with CDC (Centers for Disease Control and Prevention) stage IV (acquired immunodeficiency syndrome or AIDS) with dual infection of HIV and HTLV-1/2 (16). HIV-1 is also known to influence the outcome of HTLV-1 infection by increasing the lifetime risk of TSP/HAM in co-infected patients (17).

Fortunately, there has been a decrease in co-infection of these retroviruses, as observed in Pará, Brazil, where the prevalence of dual infection with HTLV-1/2 and HIV-1 has decreased from 8% in the 1990's to 1.4% in 2020 (18). Sex with multiple partners remained as the principal risk factor for transmission of both viruses throughout the years, denoting the role of sexual transmission in the HTLV-1 infection (18).

Holmes mentions, in his book on sexually transmitted diseases (19), that an increase in the prevalence of HTLV-1 consistent with age can be observed in all the populations studied. He reports the presence of sexual transmission in married couples and sexually active populations (sexual workers and homosexual and bisexual men), states that STIs are risk factors that increase the transmission of this virus and that infection from woman to man requires regular sexual exposure long-term. Additionally, he comments on the Peruvian studies of HTLV-1 in FSW (19), many of which revolve around the efficacy of contraception use in preventing infection.

## Sexual Transmission of HTLV-1 in PERU

Various studies performed in Peru highlight the magnitude of the sexual transmission of HTLV-1 and the need for prevention; since its prevalence is higher in FSW than in the general population. In a study of cervical secretions of seropositive FSW, virus shedding in genital tract secretions was associated with mucopurulent cervicitis (OR 4.6; 95% CI; 1.8–10.1) and gross and visible vaginal secretions (OR 2; 95% CI; 1.2–3.4) (20). No significant association between cervical shedding of HTLV-1 and gonococcal or Chlamydia infection (AOR 1.4; 95% CI, 0.6–3.2) was found. Out of 30 patients, 24 presented with bacterial vaginitis, 12 with candidiasis, and 2 with trichomoniasis; but these were not associated with HTLV-1 secretion (20). The transmission of HTLV-1 from women to men could increase due to the presence of STIs and the amount of viral load secreted, as is the case with HIV-1 (20). This would mean the treatment and prevention of STIs with the use of condoms could reduce transmission.

An early study carried out in FSW from Callao and Iquitos, documented a significantly higher prevalence of HTLV-1 in this group, which increased consistently with age, compared to a control group of prenatal clinic patients (21.8% vs. 3.1%; *p* < 0.0001). In addition, a significant association between the virus and duration of prostitution (OR 1.34 in 5-year increments; 95% CI; 1.06–1.69; *p* < 0.016) was found (21). An analysis, including controls, indicated that HTLV-1 positivity was higher in subjects with syphilis (*p* < 0.0001) and HBV markers (*p* < 0.0001). Independent associations between the virus and having been a FSW in Callao (OR 6.68; 95% CI; 3.34–11.58; *p* < 0.001), older age (OR 1.19; 95% CI; 1.04–1.36; *p* < 0.009) and positive RPR/FTA (OR 1.78; 95% CI; 1.05–3.04; *p* < 0.034) were observed (21). Once adjusted for age and occupation as FSW, the analysis identified a significant association between HTLV-1 and HBV (OR 2.05; 95% CI; 1.25–3.35) (21). The relationship between the infectious mechanisms of both viruses and the greater exposure to infection of STIs and time working as a FSW highlight the importance of sexual transmission for HTLV-1 dissemination (21).

Studies in MSM in Peru are scarce. One of the largest, looked for the presence of retrovirus in 2,655 subjects, finding HTLV-1 in 1.8%, HTLV-2 in 1.1% and HIV in 12.4% (22). In 4.6% of HIV positive patients coinfected with HTLV-1, the risk of infection was significantly associated with older age (*p* < 0.000), homosexual orientation (*p* < 0.012), receptive role (*p* < 0.021), considering themselves a sex worker (*p* < 0.012), the number of male partners (*p* < 0.047), syphilis (*p* < 0.004) and HSV-2 (*p* < 0.000) (22). Meanwhile, in those with only HTLV-1, the virus was significantly associated with older age (*p* < 0.000), having only male sexual partners (*p* < 0.030), being receptive (*p* < 0.007), anorectal abnormalities (*p* < 0.002), syphilis (*p* < 0.000) and HSV-2 (*p* < 0.000). Once the multivariate regression analysis was done, HTLV-1 was found to be associated with syphilis (AOR 2.2; 95% CI; 1.2–4.1), HSV-2 (AOR 7.7; 95% CI; 2.6–22.8), coinfection with HIV (AOR 2.6; 95% CI; 1.4–4.8), coinfection with HTLV-2 (AOR 2.9; 95% CI; 1.1–8.4) and older age (AOR 1.1; 95% CI; 1.0–1.1) (22). The authors deduced that the relationship of HTLV-1 with bisexuality and self-perception as a sex worker could lead to greater contact with FSW networks, which tend to have a higher prevalence of HTLV-1 (22).

**Figure 1:**
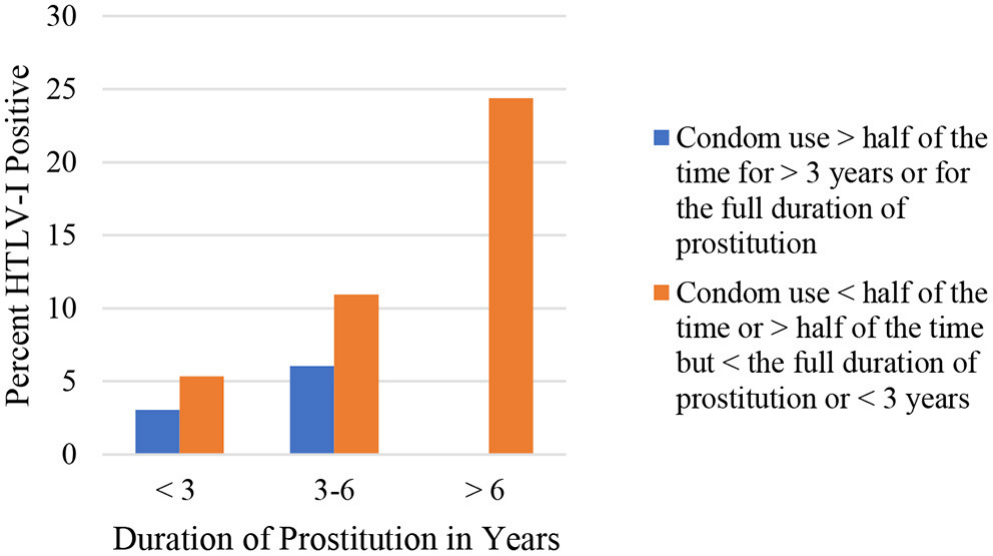
Seroprevalence of HTLV-1 according to duration of prostitution and condom use, Gotuzzo et al. (24).

**Figure 2:**
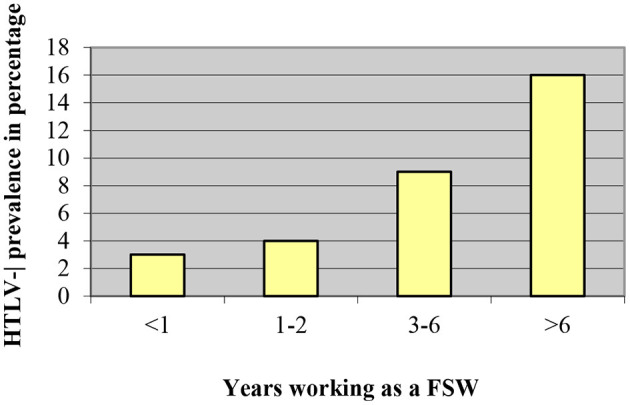
HTLV-1 prevalence according to time working as a FSW, Gotuzzo et al. (24).

It should be emphasized that the increase in condom use by sex workers in Lima and Callao during the 1990's was related to the decrease of HTLV-1 seropositivity from 21.8% in 1987–1988 to 8.7% in 2002 (20). FSW from this area reported a significant increase in condom use from 57.7% in 1993 to 84.4% in 2010 (*p* < 0.01), parallel to a significant decrease in HTLV-1 prevalence from 14.5% in 1993 to 3.1% in 2010 (*p* < 0.01) (23). A study in FSW from Lima significantly associated HTLV-1 seropositivity with condom use by women: those who were in prostitution for more than 3 years and had used a condom with more than 50% of their sexual partners and those who had been FSW for <3 years and used condoms with all their partners (OR 0.34; 95% CI; 0.13–0.89; *p* < 0.05) (Figure 1). Time working as FSW (Figure 2) and a history of infection with Chlamydia trachomatis (OR 3.8; 95% CI; 1.3–11.3; *p* < 0.02) were also significantly associated with HTLV- 1 (24). Similarly, non-significant associations were found between a higher prevalence of HTLV-1 and the practice of oral and anal sex (24).

Another study in clandestine sex workers in Lima documented the decrease of infection rate of HTLV-1 from 10.3% to 1.7% thanks to the use of condoms with all sexual partners instead of occasionally (OR 0.15; 95% CI; 0.03–0.86) (25). Likewise, a significant reduction in the history of undiagnosed STIs, genital ulcers, and inguinal adenopathy was observed due to constant condom use (25).

HTLV-1 also affects both genders of the general population. A study in asymptomatic women from three regions of Peru determined a prevalence of HTLV-1 of 2.5% (26). First sexual intercourse before the age of 20 (OR 6.4; *p* < 0.04), having had more than four sexual partners (OR 8.2; *p* < 0.04), more than four pregnancies (OR 3.1; *p* < 0.03), age ≥38 years (OR 3.8; *p* < 0.03) and <7 years of studies (OR 5, 5; *p* < 0.02) (26) were defined as risk factors for infection. This could be due to a cumulative effect caused by increased exposure to one seropositive partner for a longer time or to more sexual partners over the years; pointing out sexual transmission as the main route of dissemination. It was found as well that half of the seropositive patients were born in the Andean region and that having a father or mother with HTLV-1 was a significant risk factor (*p* < 0.05) in one of the regions evaluated (26); reaffirming this area as endemic for the virus.

Genital ulcers, sexual relations with prostitutes in the case of men, not using a condom, and a large number of sexual partners are also risk factors in the general population (7, 27–29). Likewise, the presence of the virus isolated in mononuclear cells from semen and cervical secretions (7, 30), the percentage of stable couples infected (45–55% of couples were infected in a family study, but the rate of infection was 60–75% if the index case was male) (31), the existence of dual infection of HTLV-1/2 and HIV (18.0%) (16) and the association of HTLV-1 with human papillomavirus (HPV) in Shipibo-Konibo indigenous Peruvians (32) are data that may indicate the impact of sexual transmission of HTLV-1 in Peru.

## Discussion

The low relevance given to the sexual transmission of HTLV-1 worldwide led HTLV experts from the Global Virus Network to write an open letter to the World Health Organization (WHO) in 2018 (33, 34), where they explained that 80% of infections are caused by sexual transmission and mostly affect women (source of vertical transmission). They proposed a prevention plan, which consisted of HTLV-1 screening at sexual health clinics, with follow-up of seropositive patients, notification to their partners and promotion of condom use. Donor screening; prenatal screening; provision of free safe needles; and access to an up-to-date WHO HTLV-1 database, available to all, were also proposed (33, 34).

Consequently, supported by the Australian and Japanese governments, the WHO organized a meeting in Japan, where it where it convened international experts on HTLV-1 (including from Peru) to consolidate current knowledge about the virus and how to prevent it (35). In March 2021, the conclusive reports were made public, marking a milestone in the history of HTLV-1 by recognizing it as a global public health problem and accentuating the need for preventive measures (35). However, these reports do not go into detail about the prevention of sexual transmission, given that the respective evidence is limited to observational studies on specific preventive interventions and that “there are no reports related to the prevention of sexual transmission (…)” (35). This information is incomplete, since the symposium only included controlled studies and not series or case reports, or other types of observational studies, such as those discussed here.

This article presents evidence that shows that HTLV-1 infection is more frequent in high-risk populations and that sexual transmission is greater from men to women. Likewise, the presence of other sexual infections and bacterial vaginosis favor this type of transmission, not to mention that it is consistent with time practicing as sexual workers (mostly heterosexual women). Condom use can reduce this transmission and is an inexpensive measure available to all. Nonetheless, HTLV-1 is not mentioned in the STI Treatment Guidelines of 2021 of the Center for Disease Control, be it for prevention, screening or treatment.

HTLV-1 screening is usually performed in blood and organ donors and diagnosis is reserved for those with a positive result or symptoms and risk factors which require molecular confirmation. The absence of commercially available tests of such nature and of (reliable) register systems for infected patients make the mapping of the actual dissemination of HTLV-1 difficult (36). This occurs in Brazil, in spite of the fact that they include antenatal screening in some states. Nevertheless, this country provides an example on the prevention of HTLV-1 in some areas (36).

Ideally, sexual partners of seropositive patients should be screened for infection and referred for counseling and follow up if they have a positive result, but the lack of knowledge of this virus among the population and health professionals restricts this procedure (36). Although the Brazilian government has not actively participated in the prevention of the sexual transmission of HTLV-1, numerous initiatives were developed by academic and non-governmental groups and organizations (Research Support Center on Retroviruses of the University of São Paulo, the Hemominas Foundation Journals on HTLV infection, the HTLVida Association and the Vitamoré Group – Association of HTLV carriers) looking to disseminate information about it (36).

This review highlights the lack of information on the sexual transmission of HTLV-1, the risk different sexual practices pose to the infection, and its prevention. This should be of great interest, because no appropriate treatment for the disease it causes exist and the creation of a vaccine is not yet affordable. The authors hope the information presented helps to encourage health workers and institutions responsible for the care of the population to further investigate and participate in HTLV-1 sexual transmission prevention, promoting simple and cheap methods such as adequate counseling on sexual and reproductive health and the use of condoms. It may take a long time to introduce national measures, but the awareness of health professionals can help spread prevention initiatives among local institutions and speed up the creation of adequate country and worldwide programs.

## Data Availability Statement

The original contributions presented in the study are included in the article/supplementary material, further inquiries can be directed to the corresponding author/s.

## Author Contributions

All authors participated in the conception, design, data recollection and writing of this article. The supervisor gave the final approval of the manuscript. All authors contributed to the article and approved the submitted version.

## Conflict of Interest

The authors declare that the research was conducted in the absence of any commercial or financial relationships that could be construed as a potential conflict of interest.

## Publisher's Note

All claims expressed in this article are solely those of the authors and do not necessarily represent those of their affiliated organizations, or those of the publisher, the editors and the reviewers. Any product that may be evaluated in this article, or claim that may be made by its manufacturer, is not guaranteed or endorsed by the publisher.
